# A Novel *igf3* Gene in Common Carp (*Cyprinus carpio*): Evidence for Its Role in Regulating Gonadal Development

**DOI:** 10.1371/journal.pone.0168874

**Published:** 2016-12-21

**Authors:** Feibiao Song, Lanmei Wang, Wenbin Zhu, Jianjun Fu, Juanjuan Dong, Zaijie Dong

**Affiliations:** 1 Wuxi Fisheries College, Nanjing Agricultural University, Wuxi, Jiangsu, China; 2 Freshwater Fisheries Research Centre of Chinese Academy of Fishery Sciences, Key Laboratory of Freshwater Fisheries and Germplasm Resources Utilization, Ministry of Agriculture, Wuxi, Jiangsu, China; Chinese Academy of Sciences, CHINA

## Abstract

Since the insulin-like growth factor 3 (*igf3*) gene was recently discovered in fish ovary, its function in the gonads has received much attention. In this study, we isolated two *igf3* subtypes from common carp (*Cyprinus carpio*), which comprised full-length cDNA of 707 and 1153 nucleotides encoding 205 and 198 amino acids (aa), respectively. The Igf3 aa sequence had the highest gene homology of 72% with the corresponding sequence in zebrafish (*Danio rerio*). Phylogenetic tree construction revealed that the *C*. *carpio igf3* gene was first clustered with *D*. *rerio* and then with other teleost species. *Igf3* mRNA was widely expressed, with expression being highest in the gonads and blood. In the gonad development stage, *igf3a* mRNA expression was highest in the maturity and recession stage of the ovary, and decline phase of the testis, while *igf3b* was highest in the recession and fully mature periods of the ovaries and testes, respectively. Western blotting of testis protein samples showed two bands of approximately 21 kDa and 34 kDa corresponding to the calculated molecular mass of the two Igf3 subtypes; no signal was detected in the ovary. The Igf3 protein was localized in the ovary granulosa cells and testis spermatogonium and spermatids. 17β-Ethinylestradiol treatment increased both ovary and testis *igf3* mRNA expression. These findings suggest that Igf3 may play an important role in *C*. *carpio* gonadal development.

## Introduction

Reproduction and growth are the most basic characteristics of living organisms; they are closely related but are distinguishable from each other [1]. Insulin-like growth factors (IGFs) are key factors that regulate the growth and reproduction axis [2]. IGFs consist of three ligands (IGF1, IGF2, and most recently, IGF3), two receptors (IGF1R, IGF2R), and six IGF-binding proteins (IGFBPs). In fish, the basic function of Igf1 and Igf2 is to promote growth, but significant attention has been focused on their function in fish gonad development since Igf3 was discovered in fish ovary [3,4].

Igf3 has only been reported in zebrafish (*Danio rerio*) [3,4], tilapia (*Oreochromis niloticus*) [3,5,6], and orange-spotted grouper (*Epinephelus coioides*) [7]. Two transcripts of the *igf3* gene have been found in *D*. *rerio*, predominantly expressed in the ovary: transcript variant 1 is exclusively expressed in the gonads; transcript variant 2 is only expressed during early development [4]. In teleost fish, Igf1 and Igf2 are widely expressed in various tissues; however, recent studies have shown that the *igf3* gene is specifically expressed in the gonads and brain tissue [3,7], beginning in the early stage of sex determination and differentiation [3], and is related to gonad and reproductive development [8]. *O*. *niloticus*, *D*. *rerio*, and medaka (*Oryzias latipes*) Igf3 also have the characteristic B, C, A, D, and E domains of Igf1 and Igf2, but the gene homology is relatively low [3]. Current research suggests that Igf1 and Igf3 gradually increase in the follicle cells in *D*. *rerio* [4,9], and Igf2 expression is kept at a constant level in *D*. *rerio* [10]. Igf3 expression in *D*. *rerio* strongly regulates the gonadotropin analogue and human chorionic gonadotropin (hCG) [11], and follicle-stimulating hormone (FSH) stimulates spermatogonial proliferation and differentiation in *D*. *rerio* via Igf3 [12]. While there are some relevant studies on Igf3, the function and cellular mechanism behind its regulation in fish gonad development remains unclear.

The experimental common carp (*Cyprinus carpio*) is a new aquaculture variety derived from the original parents Jian carp (*C*. *carpio* var. Jian) and Huanghe carp (*C*. *carpio haematopterus*) after one-generation mass selection and four-generation best linear unbiased prediction (BLUP) family selection by the Freshwater Fisheries Research Center (FFRC), Chinese Academy of Fishery Sciences. It is one of the dominant aquaculture species in China; its main features are high growth speed and good body shape, but the molecular mechanisms of these features are unclear [13]. In this study, the mRNA sequences encoding the *C*. *carpio igf3* gene were characterized for the first time and its temporal and spatial expression profiles in adult fish were evaluated. To understand the regulatory mechanism of *C*. *carpio* gonadal development, we located *igf3* during gonadal development and observed the effect of 17β-ethinylestradiol (E2) on its expression.

## Materials and Methods

### Animals

*C*. *carpio* were obtained from the Qiting Pilot Research Station (Yixing, China), which is affiliated with the FFRC. After adaptation, the fishes were bred at the circulation water aquaculture experimental facilities, maintained under 12-h light/dark cycles at 28 ± 1°C, and fed twice daily with special aquatic compound feed (Tech-bank Co., Ltd., Ningbo, China). This study was approved by the Animal Care and Use committee of the Centre for Applied Aquatic Genomics at the Chinese Academy of Fishery Sciences. The methods of all experiments were carried out in accordance with the Guide for the Care and Use of Experimental Animals of China.

### Fish Sampling and Tissue Preparation

At the sampling time points, fishes were anesthetized by adding clove oil (Zhanyun Chemical Co., Ltd., Shanghai, China) into the water. The fish were sacrificed to obtain the testis, ovary, blood, skin, liver, brain, muscle, intestine, kidney, spleen, and heart. The gonads (ovaries and testes) were collected in April 2014 (gonad fully mature period, stage V), July 2014 (gonad recession period, stage VI and II), October 2014 (early stage of the next gonad development cycle of fish, stage II and III), and the following January 2015 (middle stage of the next gonad development cycle of fish, stage III and IV) for gonadal expression analysis [14]. Therefore, gonad development progressed from October, January, April and July. All specimens were placed in 1.5-ml microcentrifuge tubes (KIRGEN, USA) and tissue samples were dissected out, frozen immediately in liquid nitrogen, and stored at −80°C for nucleic acid analysis.

### RNA Extraction and First-Strand cDNA Synthesis

Total RNA was extracted from the tissues using an EASY spin tissue/cell ultra-pure RNA rapid extraction kit (Yuanpinghao Biotech Co., Ltd., Tianjin, China). The concentration of total RNA was measured with a UV-spectrophotometer (NanoDrop 2000, Thermo, Wilmington, DE, USA) and the quality and integrity was checked using OD 260/280 and 1% agarose gel electrophoresis. Only samples that showed valid absorbance at the 260/280 ratio (>2.0) were used for cDNA synthesis.

The total RNA was used for the first-strand cDNA synthesis. Random 6-mers and oligo(dT) primer were used as the stem-loop reverse transcription primer; the specific procedure was performed with reference to PrimeScript RT Master Mix (Takara, Otsu, Japan). Testis RNA was used for the rapid amplification of 5′ and 3′ end cDNA (5′ and 3′ RACE) first-strand cDNA synthesis using a SMARTer RACE 5′/3′ Kit (Clontech, Mountain View, CA, USA), and then the first-strand reaction product was diluted with Tricine—EDTA buffer to 90 μL. All cDNA samples were stored at −20°C as the PCR templates.

### Igf3 Cloning and Sequence Analysis

The SMARTer RACE reaction PCR primers (Table 1) were designed based on the original expressed sequence tag (EST) sequence from the FFRC strain *C*. *carpio* transcriptome library (NCBI SRA database SRP078896). A 50-μL total reaction volume for PCR amplification was used in the SMARTer RACE nested PCR, with the specific operation performed with reference to the SMARTer RACE 5′/3′ Kit (Clontech). The PCR program proceeded as follows: 25 cycles at 94°C for 30 s, 68°C for 30 s, and 72°C for 3 min; nested PCR was performed with 28 cycles at 94°C for 30 s, 68°C for 30 s, and 72°C for 3 min in a TP600 thermocycler (Takara, Japan). The PCR products were examined with 1% agarose gel electrophoresis. Objective band size was excised with a single-use carbon steel surgical blade and purified using an E.Z.N.A. Gel Extraction Kit (Omega Bio-Tek, Norcross, GA, USA), ligated into the vector p MD18-T (Takara), transformed into *Escherichia coli* DH5-α competent cells, and sequenced.

**Table 1:** Primer sequences.

Primer	Sequences (5′–3′)	Application
*igf3* RACE F1 (first)	GCTGGCGAGGCTGCCAAAGCACG	*igf3* cloning
*igf3* RACE F2 (nested)	CCAAAGCACGCTGTGGACGAGAACT	
*igf3* RACE R1 (first)	CATCACGCTGCACCCTTTTGGGTTT	
*igf3* RACE R2 (nested)	GGAGGTCACATCCACGCACACAACA	
*igf3a* F	GGCTTGTGTTTCTGAGGCAA	Quantitative RT-PCR
*igf3a* R	TGTGTCAGTGGAAGGATGCTGT	
*igf3b* F	AGCAGCGATACCAGAAGCAT	
*igf3b* R	GAGTTCCACCGGTAAAGCGT	
β-actin F	GCTATGTGGCTCTTGACTTCGA	
β-actin R	CCGTCAGGCAGCTCATAGCT	

The resulting nucleotide and deduced amino acid (aa) sequences were analyzed using the Basic Local Alignment Search Tool (BLAST), BLASTx, and BLASTn (http://blast.ncbi.nlm.nih.gov), and the open reading frame (ORF) was predicted using Open Reading Frame Finder (http://www.ncbi.nlm.nih.gov/gorf/gorf.html). Multiple sequence alignments were generated using ClustalW2, accessed at EBI (http://www.ebi.ac.uk/Tools/msa/clustalo/). We used SMART (http://smart.embl-heidelberg.de) to estimate the cleavage site of the signal peptide; MEGA 5.0 was used to construct a neighbor-joining (NJ) phylogenetic tree, and analysis reliability was assessed by 1000 bootstrap replicates.

### Quantitative Real-Time PCR (RT-qPCR)

Tissue samples for screening the presence of *igf3* mRNA were obtained from 2-year-old sexually mature *C*. *carpio* in April 2015. The tissue distribution and changes in *igf3* mRNA expression following E2 treatment were assessed by RT-qPCR. First-strand cDNA was synthesized using PrimeScript RT Master Mix (Takara). The RT-qPCR primers were designed according to the nucleotide sequence obtained for *C*. *carpio igf3* cDNA (Table 1). *C*. *carpio* β-actin cDNA was also amplified as the internal standard (Table 1). RT-qPCR was performed with a CFX96 Real-Time PCR Detection System (Bio-Rad, Hercules, CA, USA) using SYBR Premix Ex Taq II (Takara) according to the manufacturer’s protocol. The final volume of each RT-qPCR was 25 μL, which contained 12.5 μL 2× SYBR Premix Ex Taq II, 1.0 μL diluted cDNA template (100 ng RNA), 9.5 μL PCR-grade water, and 1.0 μL of each 10 μM primer. The RT-qPCR conditions were as follows: 95°C for 30 s, followed by 40 cycles of 95°C for 5 s and 63°C for 30 s.

The *igf3* mRNA expression level in the tissues and changes in the gonads after E2 treatment were calculated using the comparative CT (2^−ΔΔCt^) method [15]. The means and standard deviations were calculated from triplicate experiments and presented as n-fold differences in expression relative to β-actin mRNA.

### Preparation of Anti—Igf3 Antibody

A synthetic peptide (GDRGFYRGKPGAARC) for Igf3 conjugated with keyhole limpet hemocyanin was emulsified with complete Freund’s adjuvant for the first immunization and with incomplete Freund’s adjuvant for the second to fourth immunizations injected into a New Zealand rabbit at 2-week intervals. Before immunization and after the third and fourth injections, the rabbit was bled and serum samples were collected. An increase in antibody titers against the peptide was verified by enzyme-linked immunosorbent assay.

### Western Blotting

Protein samples were obtained from the testis and homogenized in sodium dodecyl sulfate (SDS) sample buffer supplemented with 1 mM phenylmethanesulfonyl fluoride. Samples were analyzed using SDS-polyacrylamide gel electrophoresis (SDS-PAGE) in 12.5% (w/v) acrylamide gel with a 4% (w/v) acrylamide stacking gel using Laemmli buffer [16]. Each lane contained 10 μL gonad extract, and equal loading was confirmed by Western blot probed with an anti-β-actin (HuaAn Biotechnology Co., Ltd., Hangzhou, China) antibody. Samples were analyzed on 12.5% polyacrylamide gels under reducing conditions. After electrophoresis, the gels were electroblotted onto a polyvinylidene difluoride membrane. The membrane was treated in blocking solution (Roche, Shanghai, China) and incubated with primary antibody (1:100) for 2 h at room temperature. After washing with Tris-buffered saline (20 mM Tris-HCl, 0.9% NaCl, pH 7.4) containing 0.1% Tween-20 3 times at 10 min per wash, the membrane was incubated with goat anti-rabbit serum (1:5000) labeled with alkaliphosphatase (Bio-Rad) for 1 h at room temperature. After washing with Tris-buffered saline 3 times at 10 min per wash, detection was performed using nitroblue tetrazolium and 5-bromo-4-chloro-3-indolyl phosphate (NBT/BCIP; Roche) as the substrate.

### Immunohistochemistry

The gonads of male and female juvenile *C*. *carpio* were dissected and fixed in Bouin’s solution at room temperature overnight with gentle shaking. Tissue blocks were dehydrated in an ascending series of ethanol, cleared in xylene, embedded in paraffin, and sectioned at 5-μm thickness. The paraffin sections were deparaffinized and hydrated, followed by xylene I for 10 min, xylene II for 10 min, absolute ethyl alcohol for 5 min, absolute ethyl alcohol II for 5 min, 95% alcohol for 5 min, 90% alcohol for 5 min, 80% alcohol for 5 min, 70% alcohol for 5 min, and washed with distilled water. After 10-min incubation in 0.3% methanol with hydrogen peroxide and washing in 0.01 M PBS 3 times at 3 min per wash, the sections were immersed in 0.01 M citric acid buffer (pH 6.0) containing 0.1% Tween 20, autoclaved for 5 min, cooled at room temperature for 10 min, and washed with 0.01 M PBS 3 times at 3 min per wash. The sections were then treated in blocking solution (Roche), incubated with Igf3 rabbit monoclonal antibody (1:200) overnight at 4°C, and rinsed with 0.01 M PBS 3 times at 3 min per wash. Subsequently, the sections were incubated with horseradish peroxidase—conjugated goat anti-rabbit IgG for 30 min, and then rinsed with PBS three times at 3 min per wash. Immunoreactive signals were visualized using diaminobenzidine (Sigma) as the substrate. Sections were counterstained with hematoxylin-eosin. In the negative control, the primary antibody was replaced with normal mouse serum.

### E2 Treatment

Male and female juvenile *C*. *carpio* (6 months old) were randomly divided into 4 treatment groups and 1 control group. Three tanks were randomly assigned to each group, with 12 fishes per tank. The treatment groups were injected intraperitoneally with 1 μg/g, 5 μg/g, 10 μg/g, or 50 μg/g body weight E2 (Dr. Ehrenstorfer GmbH, Augsburg, Germany); the E2 was dissolved in an equal volume mixture of alcohol and 0.6% physiological saline. The control group was injected with identical volume of solution without E2. The injections were repeated the following day at the same time; subsequently, samples were obtained at 24 h, 48 h, and 72 h, and 3 fish samples were obtained per tank each time. Samples were snap-frozen in liquid nitrogen and stored at −80°C until RNA extraction. Three biological samples (*n* = 3) were analyzed.

### Statistical Analysis

All data are presented as the mean ± standard error of the mean (SEM). One-way analysis of variance followed by the Duncan test (SPSS Inc., Chicago, IL, USA) was performed to identify significant differences between treatment groups. The results between treatment and control groups were compared using Student’s *t*-test. Statistical significance was determined at *p* < 0.05.

## Results

### Igf3 Cloning and Sequence Analysis

**Fig 1:**
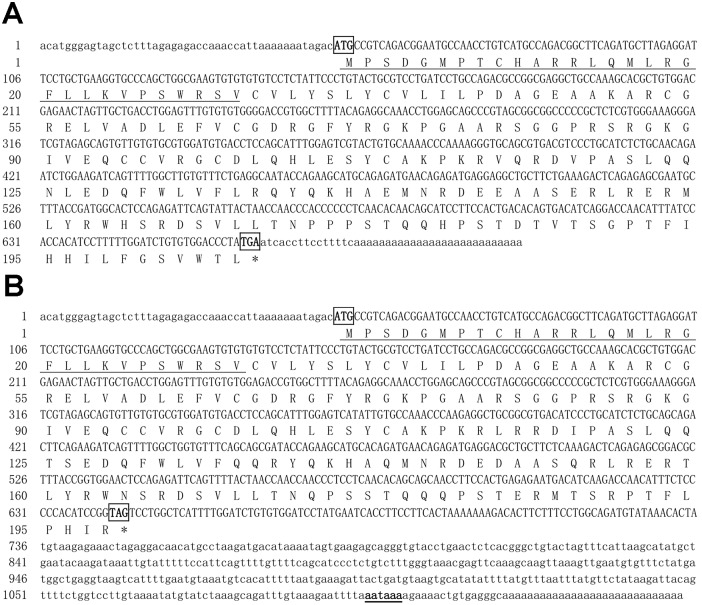
Nucleotide sequences and the deduced aa sequences of *C*. *carpio* Igf3a (A) and Igf3b (B). The start codons (ATG) and stop codons (TGA) are boxed; the putative signal peptides are underlined (A, B); underlined and bold sequences indicate the polyadenylation sites (AATAAA) (B).

In this study, two complete cDNA sequences of *igf3* were cloned from *C*. *carpio* and were designated *igf3a* (GenBank accession number: KT895499) and *igf3b* (GenBank accession number: KT895500). *Igf3a* consists of 707 nucleotides with a 618-bp ORF, a 47-bp 5′ untranslated region (UTR), and a 42-bp 3′ UTR, and encodes 205 amino acids (aa) (Fig 1A). *Igf3b* consists of 1153 nucleotides with a 597-bp ORF, a 47-bp 5′ UTR, and a 509-bp 3′ UTR, and encodes 198 aa (Fig 1B).

The *C*. *carpio* novel Igf3 sequences also had the characteristic B, C, A, D, and E domains of Igf1 and Igf2. However, amino acid comparison of the *C*. *carpio* Igf1, Igf2, and Igf3 sequences (Table 2) showed that the *C*. *carpio* Igf3a and Igf3b sequences shared low similarity with *C*. *carpio* Igf1 (20.49% and 20.71%, respectively), Igf2a (25.35% and 25.94%, respectively), and Igf2b (26.07% and 25.71%, respectively). The Igf3 B and A domains shared about 60% aa identity with Igf1, and about 50%–60% aa identity with Igf2. The D and E domains shared about 30% aa identity with Igf1 and about 42%–50% aa identity with Igf2. The C domain shared the lowest aa identity with Igf1 and Igf2, i.e., 16% and 25%, respectively.

**Table 2:** Percent identity matrix of aa pairwise comparison for *C*. *carpio igf* genes.

	Igf1	Igf2a	Igf2b	Igf3a	Igf3b
Igf1					
Igf2a	28.50				
Igf2b	32.38	52.83			
Igf3a	20.49	25.35	26.07		
Igf3b	20.71	25.94	25.71	85.37	

### Amino Acid Sequence Alignment

**Fig 2:**
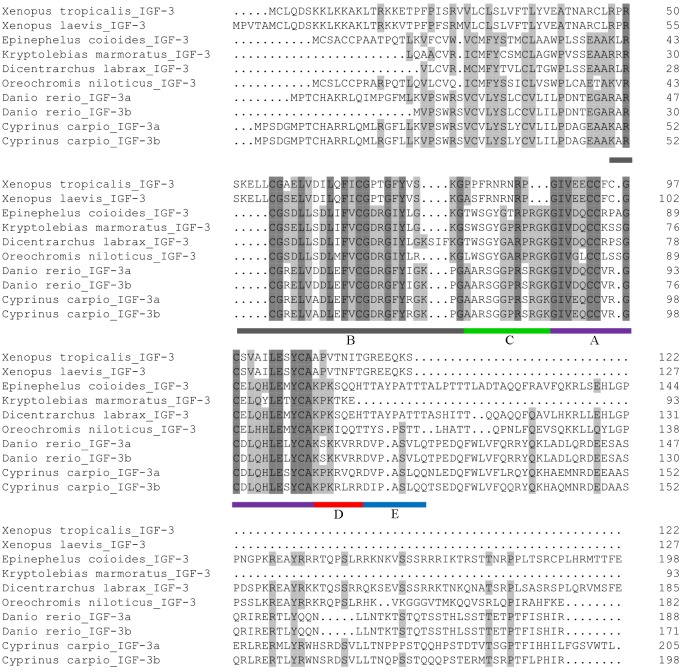
Comparison of Igf3 aa sequences from different species. Dark shaded areas indicate examples of species with the same aa sites; light shaded areas indicate that more than half of the listed species have the same aa sites. The mature region (B, C, A, D domains) and E domain are underlined with different colors.

Comparison of the *C*. *carpio* Igf3 deduced aa sequence with other vertebrate Igf3 aa sequences is shown in Fig 2. The *C*. *carpio* Igf3 deduced aa sequence had low homology to that of other teleost fishes. The *C*. *carpio* deduced aa sequence shared the highest similarity with *D*. *rerio* (72%, GenBank accession number: Igf3a: ADO16598.1, Igf3b: ADO16599.1), and shared similarity of 58% with *Kryptolebias marmoratus* (AGA82753.1), 55% with *O*. *niloticus* (NP_001266565.1) and *Dicentrarchus labrax* (AGB51126.1), and 39% with *E*. *coioides* (AML84199.1).

### Phylogenetic Analysis of Three *igf* Genes

**Fig 3:**
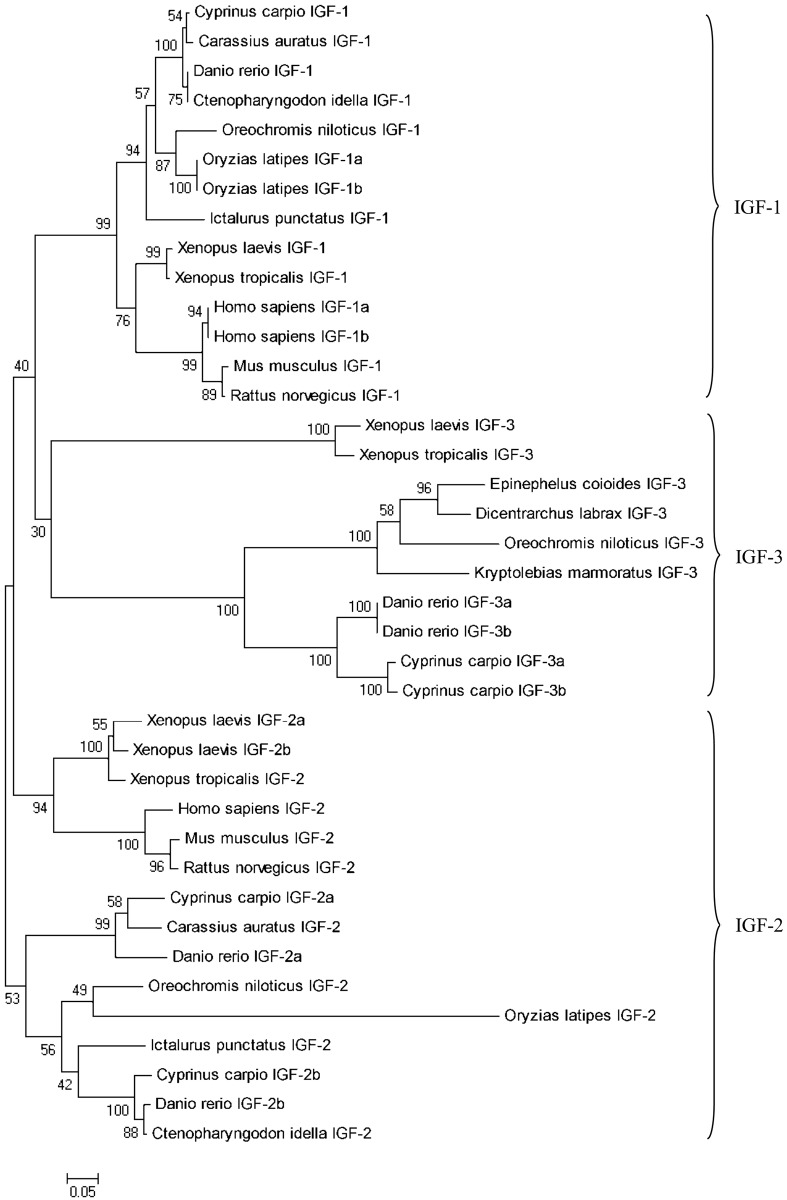
Phylogenetic analysis of Igf1, Igf2 and Igf3 in vertebrates. NJ phylogenetic tree of Igf3 using the deduced aa sequences of *C*. *carpio* and the Igf1, Igf2 and Igf3 deduced aa sequences of other species in the GenBank database: *Homo sapiens* (Igf1a: AAA52538.1, Igf1b: AAA52537.1, Igf2: AAA60088.1), *Mus musculus* (Igf1: AAH12409.1, Igf2: AAH58615.1), *Rattus norvegicus* (Igf1: P08025.3, Igf2: XP_008758297.1), *D*. *rerio* (Igf1: AAI14263.1, Igf2a: NP_571508.1, Igf2b: NP_001001815.1, Igf3a: ADO16598.1, Igf3b: ADO16599.1), *C*. *carpio* (Igf1: AKK31842.1, Igf2a: ADO16599.1, Igf2b: ADQ44896.1, Igf3: obtained in the present study), *Carassius auratus* (Igf1: AHZ62772.1, Igf2: ACJ37293.1), *X*. *laevis* (Igf1: NP_001156865.1, Igf2a: NP_001082128.1, Igf2b: NP_001085129.2, Igf3: NP_001082137.1), *X*. *tropicalis* (Igf1: XP_002936875.1, Igf2: AAI56000.1, Igf3: NP_001120418.1), *O*. *niloticus* (Igf1: NP_001266432.1, Igf2: NP_001120418.1, Igf3: NP_001266565.1), *Ctenopharyngodon idella* (Igf1: AGW17294.1, Igf2: NP_001266565.1), *Ictalurus punctatus* (Igf1: AAZ28918.1, Igf2: NP_001187128.1), *Oryzias latipes* (Igf1a: XP_011489575.1, Igf1b: XP_004083254.2, Igf2: XP_011491450.1), *Dicentrarchus labrax* (Igf3: AGB51126.1), *K*. *marmoratus* (Igf3: AGA82753.1), and *E*. *coioides* (AML84199.1). Numbers at each branch indicate the bootstrap value (%). Bar at the bottom indicates 5% aa divergence in the sequences.

Phylogenetic analysis of *igf1*, *igf2*, and *igf3* from representative fishes, amphibians, and mammals produced an NJ phylogenetic tree clustered into three separate clades. The NJ phylogenetic tree revealed that the *C*. *carpio igf3* gene was first clustered with that of *D*. *rerio* and then with other teleost fishes, finally clustering with African clawed frog (*Xenopus laevis*) and tropical clawed frog (*X*. *tropicalis*). Phylogenetic analysis also revealed that *igf3* was first clustered with *igf1* and then with *igf2*, indicating that *igf3* is more closely related to *igf1* (Fig 3).

### Expression Pattern of *igf3*

**Fig 4:**
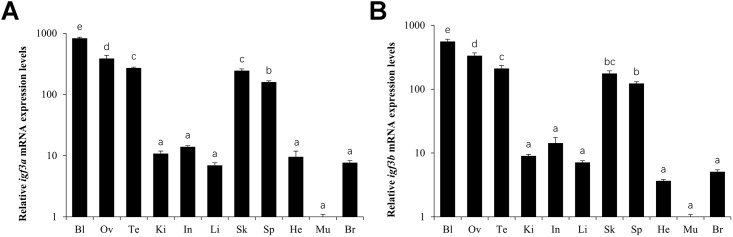
Profile of *igf3a* (A) and *igf3b* (B) mRNA expression in adult *C*. *carpio*. Blood (Bl), ovary (Ov), testis (Te), kidney (Ki), intestine (In), liver (Li), skin (Sk), spleen (Sp), heart (He), muscle (Mu), brain (Br).

RT-qPCR examination of the tissue expression of the *C*. *carpio igf3* gene showed that *igf3a* and *igf3b* was widely expressed and share similar expression pattern, with expression being highest in the blood and gonads. *Igf3* mRNA expression was weaker in the skin, spleen, intestine, kidney, liver, brain, and heart, and expression was lowest in the muscle (Fig 4).

**Fig 5:**
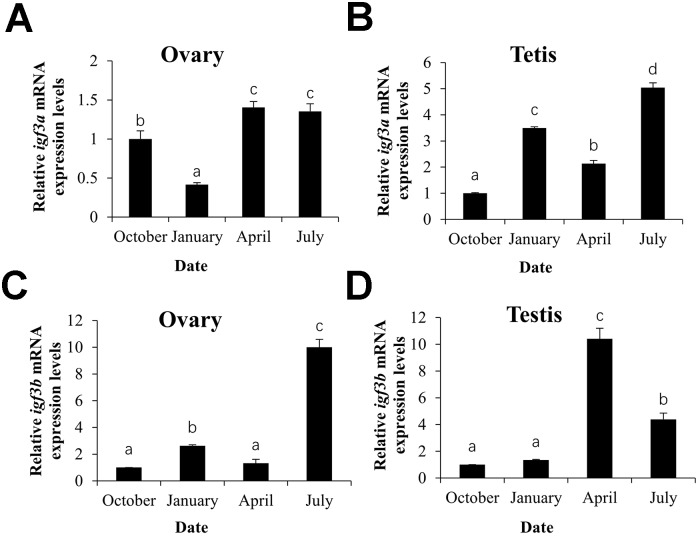
Expression of *C*. *carpio igf3a* (A, B) and *igf3b* (C, D) mRNA in gonadal development.

The expression of *igf3a* mRNA was first decreased in middle stage of the next gonad development cycle of fish (January) (*p* < 0.05), then peaked in the fully mature period (April) (*p* < 0.05) as the ovary development progressed, but there was no significant change in the ovaries recession period (July) (*p* > 0.05, Fig 5A). Nevertheless, *igf3a* mRNA in the testis was first increased (*p* < 0.05) and then decreased (*p* < 0.05) in the fully mature period (April) (*p* < 0.05), but peaked in the testes recession period (July) (*p* < 0.05, Fig 5B). The *igf3b* mRNA expression pattern in the ovary was similar with *igf3a* in the tetis (Fig 5C). *Igf3b* mRNA expression in the testis was increased in October (early stage of the next gonad development cycle of fish) and the following January (*p* > 0.05), peaked in April (*p* < 0.05), and then decreased in July (*p* < 0.05, Fig 5D).

### Western Blot Analysis

**Fig 6:**
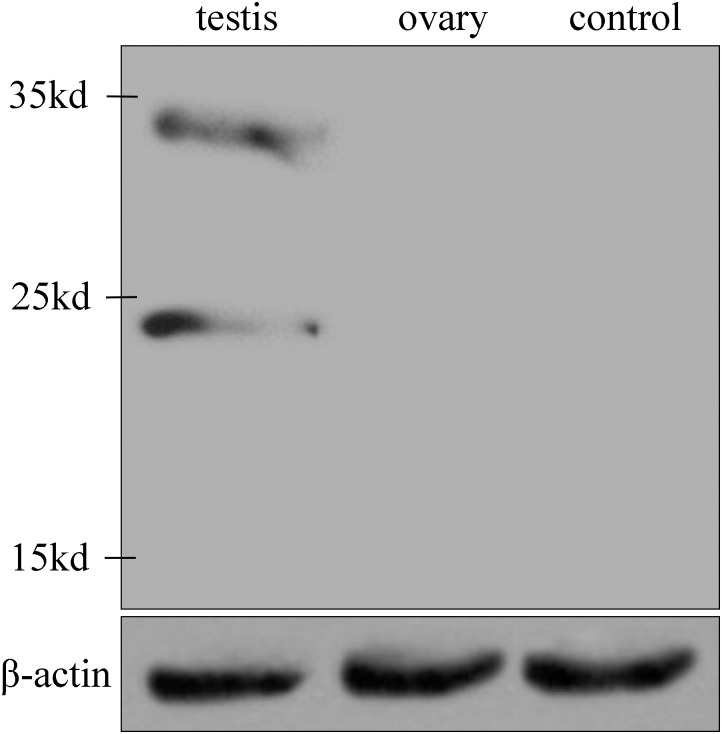
Western blot analyses of Igf3 from *C*. *carpio* testis and ovary.

Two specific bands characteristic of Igf3 were observed on the immunoblots when the protein extracted from the *C*. *carpio* testis was transferred to a nitrocellulose membrane and probed with anti-Igf3, with no signal detected at the ovary (Fig 6). The anti-Igf3 recognized bands of approximately 21 kDa and 34 kDa, which corresponded with the calculated molecular mass for the *C*. *carpio* Igf3 subtypes. Control serum from the pre-immunized rabbit did not recognize any protein component in the *C*. *carpio* extracts (Fig 6).

### Localization of Igf3 in the Testes and Ovaries

**Fig 7:**
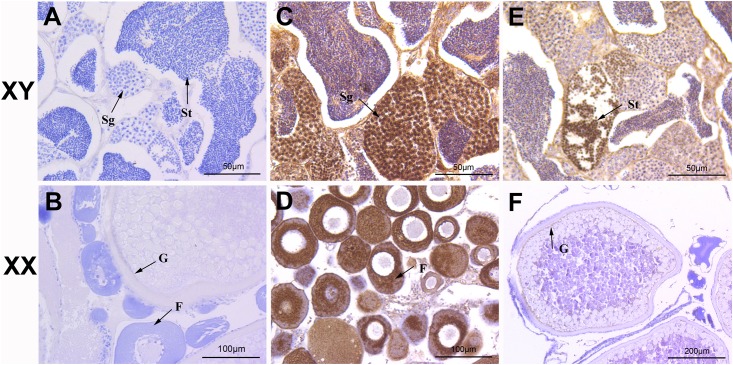
Immunohistochemistry localization of Igf3 in *C*. *carpio* testis and ovary. Positive signals of anti-Igf3 immunolabeling are shown in brown. Igf3 was expressed in the spermatogonium (**C)** and spermatids (**E**)of the testis. Igf3 was expressed in the follicle cells (**D**) and granulosa cells of the ovary (**F**). A negative control for the testis (**A**) and ovary (**B**). Sg, spermatogonium; St, spermatids; F, follicle cell; G, granulosa cell.

Localization of *C*. *carpio* Igf3 expression in the gonads was determined using immunohistochemistry. Immunoreactive positive signals (brown) for Igf3 were detected in both the testis and ovary. In the testes, the signals became stronger as the sperm cell development progressed. Igf3 was mainly concentrated in the spermatogonium and spermatids of 10-month-old *C*. *carpio* testis (Fig 7C and 7E). The signals became weaker as oocyte development progressed. In the ovary, Igf3 was concentrated mainly in the follicle cells in the early development stage (Fig 7D) and mainly in the granulosa cells in the late development stage (Fig 7F). No positive signal was found in the negative control (Fig 7A and 7B).

### Effect of E2 on *igf3* mRNA Expression

**Fig 8:**
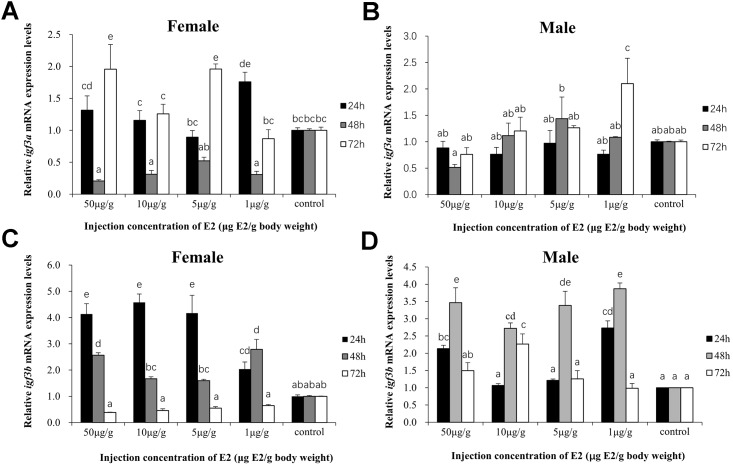
Effect of E2 on *igf3* mRNA expression levels in the testis and ovary. Y-axis shows data presented as the mean ratios ± SEM between treatment and control groups.

RT-qPCR showed that different doses and durations of E2 treatment changed the relative *igf3* mRNA expression level in the testis and ovary (Fig 8).

*Igf3a* mRNA expression in the ovary was significantly increased at 24 h (*p* < 0.05) of 1 μg/g E2 group, and all treatment groups decreased to a minimum at 48 h; Nevertheless, increased again at 72 h (Fig 8A). In the testis, *igf3a* mRNA expression levels were not significantly different between different doses and durations of E2 treatments and control groups (*P* > 0.05) except 1 μg/g E2 group at 72 h (*p* < 0.05, Fig 8B). *Igf3b* mRNA expression peaked at 24 h (*p* < 0.05) in the ovary, and then decreased (Fig 8C). In the testis, a significantly increase in the expression of *igf3b* only after 48 h of incubation (*p* < 0.05), then decreased at 72 h (Fig 8D).

## Discussion

The genome was duplicated in the long evolutionary process of teleost fish [17–19]. In this study, two distinct full-length cDNA subtypes of the *igf3* gene, which is involved in fish gonad development, were characterized in *C*. *carpio* for the first time. Interestingly, the two subtype sequences had the same 5′ UTR, but different 3′ UTRs, which resulted in the same transcription initiation sites and signal peptides (Fig 1). To date, only *C*. *carpio* and *D*. *rerio* have been found to have two *igf3* mRNA sequences; other teleost fish only have one *igf3* subtype [3,7]. This may indicate that *C*. *carpio* and *D*. *rerio igf3* was highly conserved during species evolution, and different genome duplications in teleost fish caused the evolutionary diversity of *igf3*. Characterization of the *C*. *carpio igf3* gene revealed that it has the typical characteristics of the Igf family, including the five typical domains and six conserved cysteine residues. It also shares a similar tertiary protein structure to *igf1* and *igf2* despite sharing relatively low sequence homology with them, a finding similar to *D*. *rerio* and *O*. *niloticus* [3]. Phylogenetic analysis showed that the *C*. *carpio igf3* was clustered into clades independent of *igf1* and *igf2*, further evidence that Igf3 is a novel member of the Igf family.

RT-qPCR showed that, in *O*. *niloticus*, *igf3* transcripts were distributed in various tissues except the muscle and kidney, and high levels were found in the testis and ovary [5]. In *E*. *coioides*, *igf3* transcripts were detected in the ovary and areas of the brain, especially the telencephalon and mesocerebrum [7], but were detected only in the testis and ovary tissues in *D*. *rerio* [3,11] when examined by RT-PCR. In this study, *igf3* transcripts were found in all tissues of *C*. *carpio*, and they share similar expression pattern; *igf3* expression was highest in the blood, and its expression levels were lower in the testis and ovary (Fig 4). The observed tissue distribution patterns might be the result of the higher sensitivity of RT-qPCR over RT-PCR and the use of different species. The different expression levels in the gonads in the different species are probably due to the samples being obtained at different developmental stages. However, the *igf3* mRNA levels were consistently high in the ovaries and testes, indicating that the *igf3* gene plays an important role in gonadal development in teleost fish. *Igf3* mRNA is expressed in the tissues of the hypothalamic-pituitary-gonadal axis and the ovary [7], which suggests involvement of the hypothalamic-pituitary-gonadal axis. We also found high expression of *igf3* mRNA in adult *C*. *carpio* blood, and we speculate that the blood is involved in transporting hypothalamic-pituitary-gonadal axis secretions [20].

The detailed spatio-temporal expression profiles of the *C*. *carpio igf3* gene were further investigated during gonad development. In *D*. *rerio*, *igf3* expression increased significantly as follicles developed [4], but another study found that it significantly decreased in the hours prior to ovulation and was confined to the follicle cells [11]. This may mean that ovarian *igf3* mRNA expression is related to age in teleost fish [2,21]. However, in *E*. *coioides*, *igf3* mRNA expression is not significantly different during ovarian development [7]. In this study, *igf3a* mRNA relative expression was highest in the fully mature period (April) and ovaries recession period (July), Nevertheless, *igf3b* was only highest expressed in July, but low inApril. *C*. *carpio* yolk accumulation ends in April, the *igf3a* mRNA expression was increased, but *igf3b* was decreased, which suggesting that *igf3* may participate in yolk accumulation and *igf3a* may participate in ovulation. In July, the *C*. *carpio* ovary degenerates and proceeds to next development, *igf3a* may had the same effect as *igf3b* at this stage. In the *C*. *carpio* testis, the increasing level of *igf3a* mRNA in January and July indicating that *igf3a* may plays an important role during fish testis differentiation and development. *igf3b* mRNA expression was highest in April but decreased in July, which was opposite to that of *igf3a*. This may mean that the *igf3b* gene not only affects spermatogenesis and maturity [12], but may also affect sperm release [22], and the modes of Igf3a and Igf3b regulation in the testis are quite different. The *igf3* mRNA expression levels differing as gonadal development progressed in *C*. *carpio* may have been due to the asynchronous development of eggs and sperms, and the samples may have contained eggs and sperm at different developmental stages.

Western blotting revealed two specific bands of approximately 21 kDa and 34 kDa in the protein samples extracted from the testis that corresponded to the calculated molecular weights of the two Igf3 subtypes, and were similar to that of *O*. *niloticus* His-Igf3 and GST-Igf3 recombinant protein [6]. Whereas, no signal was detected in the ovary. This may indicate that the major biological action of Igf3 in *C*. *carpio* is the regulation of testicular functions. In *O*. *niloticus*, *in situ* hybridization determined that Igf3 is expressed in the ovarian follicle cells of 2-month-old fish and mainly in the granulosa cells of the adult ovary, and in the spermatogonium and spermatids of the testis [3]. Immunohistochemistry revealed that *C*. *carpio* Igf3 was concentrated mainly in the spermatogonium and spermatids in the testis, and in the follicle cells and granulosa cells of the early and late developmental stages, respectively, in the ovary. Igf3 expressed in the spermatogonium and spermatids of the testis may play an important role in promoting sperm formation, and its expression in the follicle cells and granulosa cells of the ovary may promote oocyte development and maturation.

Vertebrate reproductive activity is mainly affected by regulation of the hypothalamic-pituitary-gonadal axis [23]. Estrogen, especially E2, plays an important role in the growth and development of female animals, especially in gonad development, sex regulation, and reproductive behavior. E2 is involved in promoting vitellogenesis and follicle growth during the early stages of ovarian development [11]. Previous studies have shown that, in adult fish, E2 modifies expression of the components of the Igf system [24–26], and treatment with 17α-estradiol (EE2) during ontogeny will persistently impair the development and growth of fish and organs [27–29]. In this study, intraperitoneal E2 could enhanced ovary Igf3a expression in juvenile *C*. *carpio*, but only in 50 μg/g 72h, 10 μg/g 72h and 1 μg/g 24h. In testis, Igf3a expression levels of E2 treatments were not significantly different between control groups. This is probably due to that the Igf3a is not susceptible to estrogen regulation. Nevertheless, intraperitoneal E2 enhanced ovary Igf3b expression, and expression peaked at 24 h before beginning to markedly declined to that of control group at 72 h. Interestingly, *igf3b* mRNA expression in the testis was also enhanced after E2 treatment, peaking at 48 h, and then significantly decreased at 72 h except the 10 μg/g E2 group. This was not concordant with the results of EE2 treatment, where both feeding of high EE2 doses and exposure to low, environmentally relevant EE2 resulted in significant down-regulation of *igf3* mRNA in the testis while ovarian *igf3* mRNA was not affected [5]. This was probably due to the different species and developmental conditions, and different estrogens [30]. The *C*. *carpio igf3* gene appears to be more susceptible to estrogen regulation, and *igf3b* is more sensitive than *igf3a*. Based on the *igf3* mRNA expression ratio between the testis and ovary after the same E2 treatment, the effect of E2 differs between sexes. It may mean that, at the gonadal development stage, *igf3* mRNA in juvenile *C*. *carpio* ovary is more susceptible to estrogen challenge than the testis is [31]. In *C*. *carpio*, the testes develop earlier than the ovaries, so this may be another reason for the effect of E2. These studies have all shown that Igf3 may have a substantial effect on gonadal development in fish and that estrogen influences the expression of its gene [4,5,32].

## Conclusions

We successfully cloned two complete *igf3* cDNA sequences from *C*. *carpio*, performed sequence analysis, characterized the *igf3* mRNA distribution in various tissues, investigated the *igf3* expression pattern at different stages of gonadal development, located Igf3 in the gonads and following E2 treatments. *Igf3* mRNA was mainly expressed in the gonads; expression of *igf3a* was highest in the maturity stage and decline phase of the ovary, decline phase of the testis, *igf3b* was highest in the decline phase and maturity stage of the ovary and testis, respectively. Immunohistochemistry revealed that Igf3 was localized in the spermatogonium and spermatids of the testis and in the follicle cells and granulosa cells of the early and late developmental stages, respectively, in the ovary. E2 treatments regulated *igf3* mRNA expression.

## Supporting Information

S1 Fig Profile of *igf3* mRNA expression in adult *C*. *carpio*. Expression were studied by RT-qPCR and semi-quantitative RT-PCR.
(TIF)

S2 Fig The paraffin sections of gonadal development in *C*. *carpio*. October: early stage of the next gonad development cycle of fish, stage II and III, January: middle stage of the next gonad development cycle of fish, stage III and IV, April: gonad fully mature period, stage V, July: gonad recession period, stage VI and II.
(TIF)
