# Supplementary figures and images for: A Novel igf3 Gene in Common Carp (Cyprinus carpio): Evidence for Its Role in Regulating Gonadal Development
Source: PLoS One. 2016 Dec 21;11(12):e0168874. doi: 10.1371/journal.pone.0168874 (PMC5176323; doi:10.1371/journal.pone.0168874)

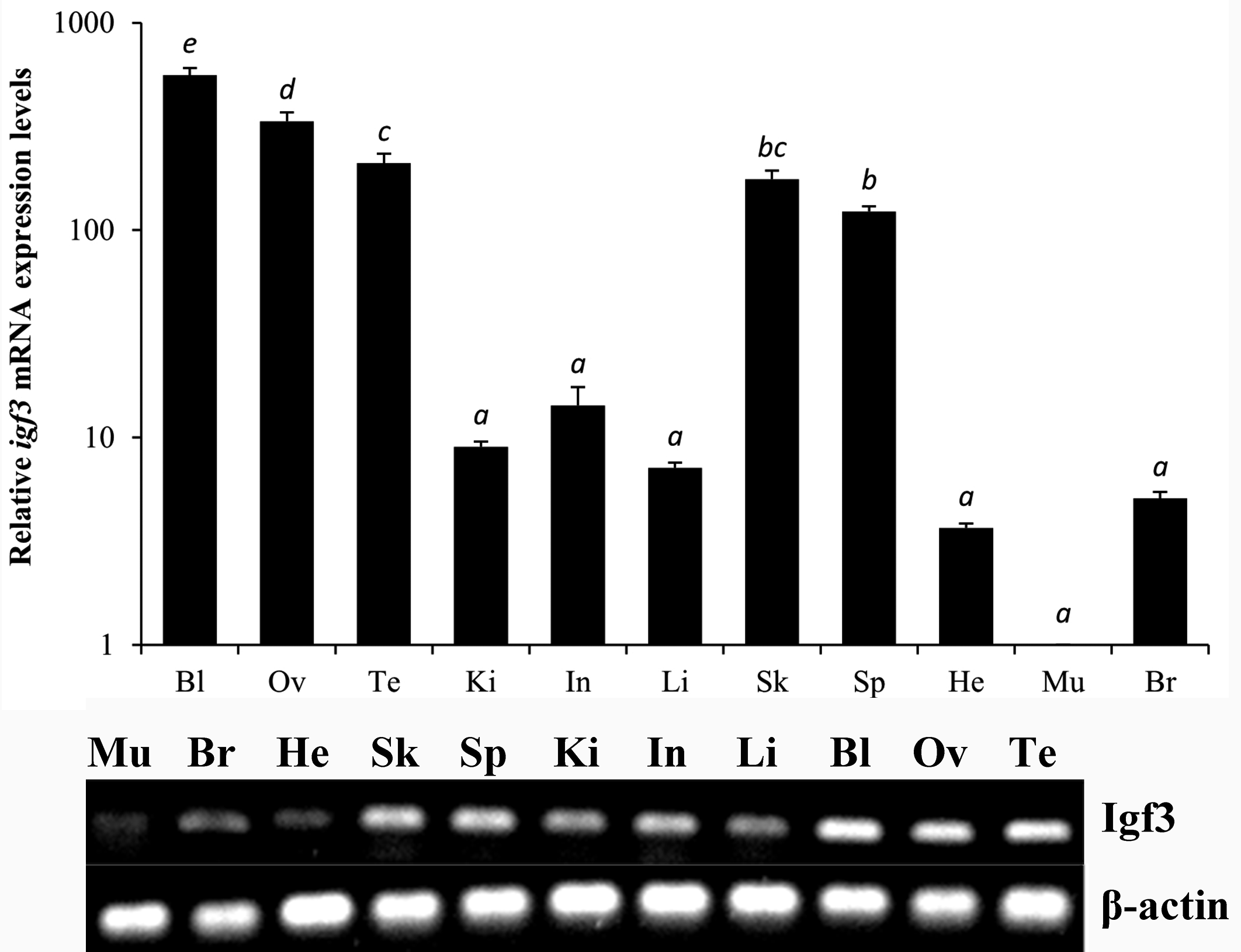

Supplement: S1 Fig — Expression were studied by RT-qPCR and semi-quantitative RT-PCR. (TIF) [file pone.0168874.s001.tif]

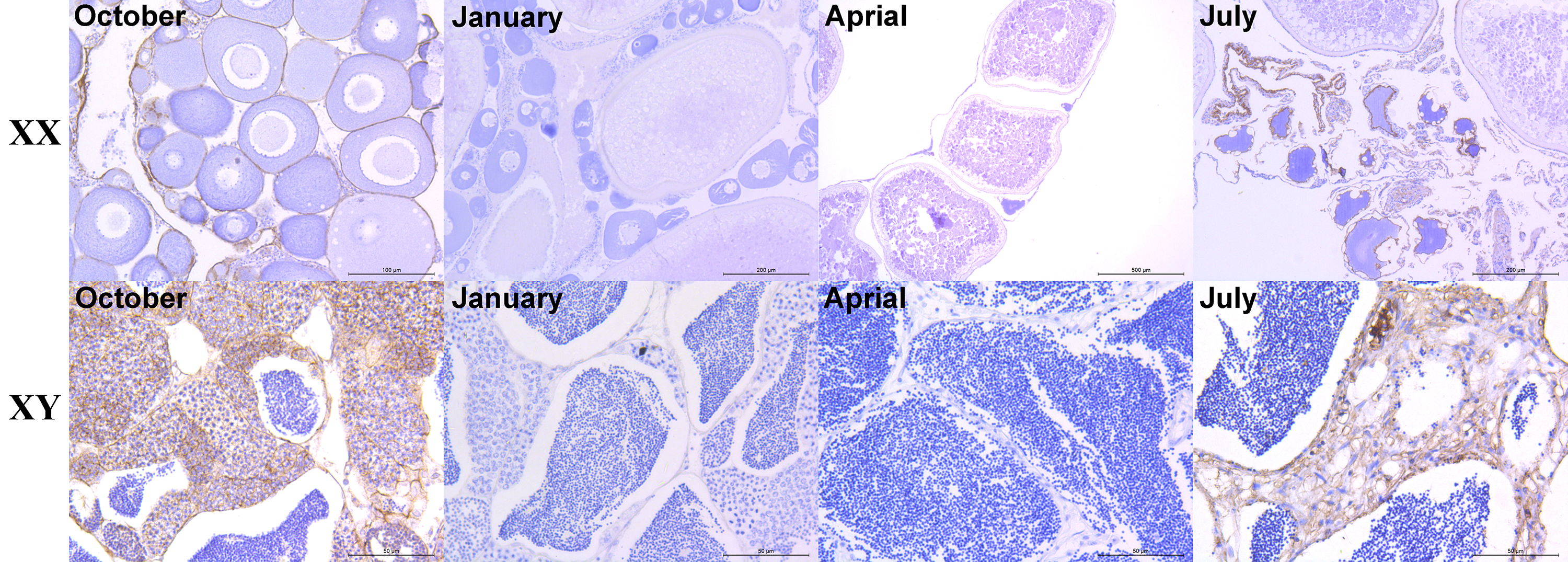

Supplement: S2 Fig — October: early stage of the next gonad development cycle of fish, stage II and III, January: middle stage of the next gonad development cycle of fish, stage III and IV, April: gonad fully mature period, stage V, July: gonad recession period, stage VI and II. (TIF) [file pone.0168874.s002.tif]
